# Activation of AQP2 water channels without vasopressin: therapeutic strategies for congenital nephrogenic diabetes insipidus

**DOI:** 10.1007/s10157-018-1544-8

**Published:** 2018-02-24

**Authors:** Fumiaki Ando, Shinichi Uchida

**Affiliations:** Department of Nephrology, Tokyo Medical and Dental University, 1-5-45 Yushima, Bunkyo-ku, Tokyo, 113-8510 Japan

**Keywords:** Congenital nephrogenic diabetes insipidus, AQP2, Calcium signaling, cAMP signaling, GPCRs agonists, PDE inhibitors

## Abstract

Congenital nephrogenic diabetes insipidus (NDI) is characterized by defective urine concentrating ability. Symptomatic polyuria is present from birth, even with normal release of the antidiuretic hormone vasopressin by the pituitary. Over the last two decades, the *aquaporin-2 (AQP2)* gene has been cloned and the molecular mechanisms of urine concentration have been gradually elucidated. Vasopressin binds to the vasopressin type II receptor (V2R) in the renal collecting ducts and then activates AQP2 phosphorylation and trafficking to increase water reabsorption from urine. Most cases of congenital NDI are caused by loss-of-function mutations to V2R, resulting in unresponsiveness to vasopressin. In this article, we provide an overview of novel therapeutic molecules of congenital NDI that can activate AQP2 by bypassing defective V2R signaling with a particular focus on the activators of the calcium and cAMP signaling pathways.

## Introduction

Congenital nephrogenic diabetes insipidus (NDI) is characterized by the increased excretion of diluted urine in spite of appropriate secretion of the antidiuretic hormone vasopressin. In severe cases, patients excrete up to 10–20 L of urine per day [1]. Excessive urine output and drinking seriously reduce quality of life and the ability to participate in social activities. Polyuria also induces structural changes to the urinary tract, which lead to the onset of chronic kidney disease. Moreover, growth and mental retardation are often found on long-term follow-up despite adequate water balance control [2]. To prevent these complications of congenital NDI, further drug discovery is required.

Loss-of-function mutations to the vasopressin type 2 receptor (V2R) are found in approximately 90% of patients with congenital NDI [3]. V2R is encoded by the *AVPR2* gene, which is located at chromosome Xq28. X-linked recessive NDI occurs in about one in 250,000 males. In Japan, an estimated 400 people have congenital NDI. In the other 10% of patients, congenital NDI has an autosomal recessive or autosomal dominant mode of inheritance with mutations to the *aquaporin-2 (AQP2)* gene [4, 5].

**Fig. 1:**
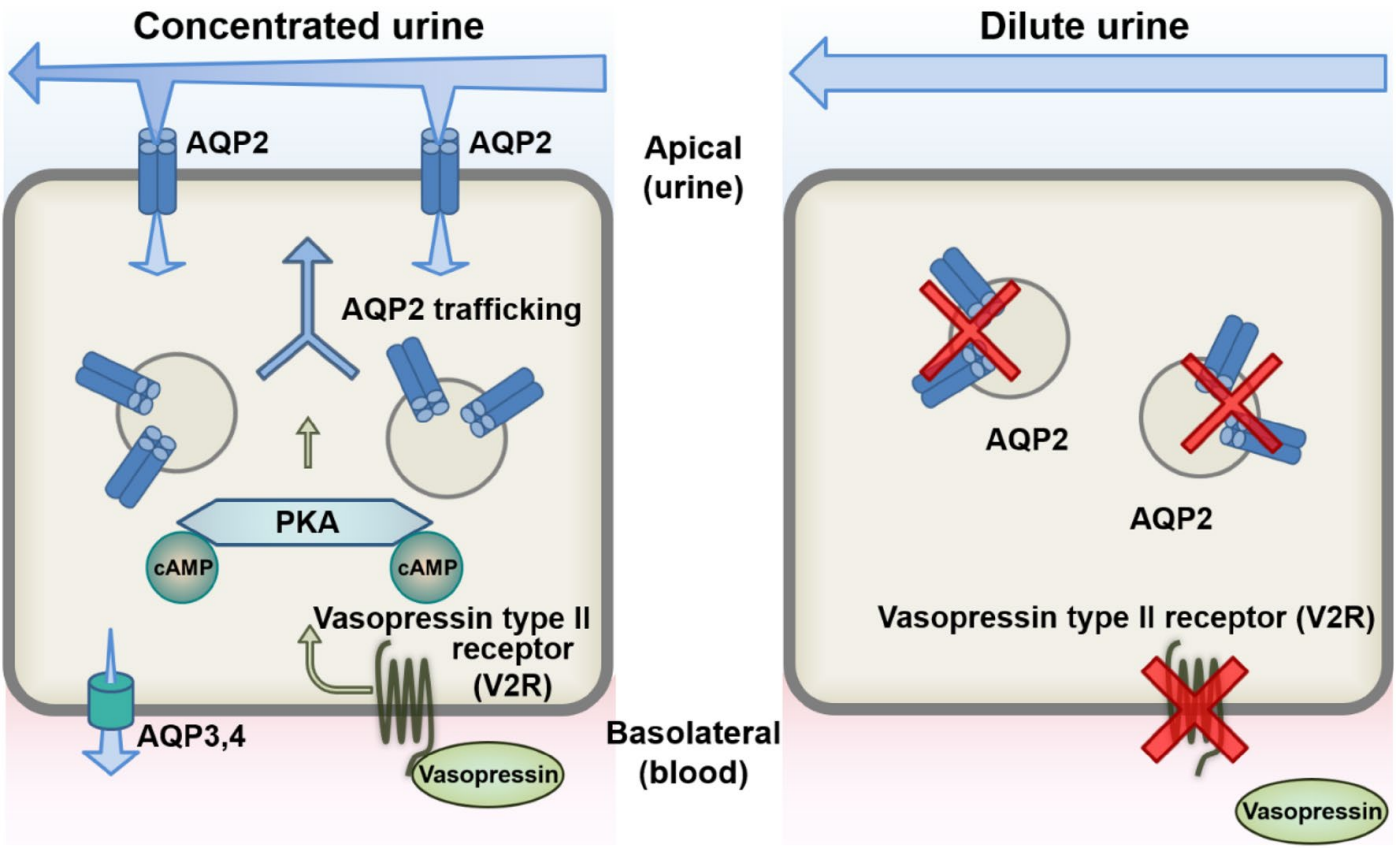
The mechanisms of urine concentration by vasopressin. (Left) Circulating vasopressin binds to V2R in the basolateral membrane of cells of the renal collecting ducts. Adenylyl cyclase is then activated and increases cAMP production and PKA activity, leading to AQP2 phosphorylation. Changes in AQP2 phosphorylation status leads to translocation of cytosolic AQP2 to the apical plasma membrane. Water is reabsorbed from urine through AQP2, AQP3, and AQP4, thereby concentrating the urine. (Right) V2R mutations account for 90% of all diagnoses of congenital NDI, while AQP2 mutations occur in the other 10%. Defective V2R or AQP2 function impairs water reabsorption, resulting in urine dilution

V2R and AQP2 are major regulators of urine concentration (Fig. 1). In response to dehydration, the antidiuretic hormone vasopressin is secreted from the posterior pituitary. Binding of vasopressin to its receptor V2R in the renal collecting ducts increases intracellular production of cyclic adenosine monophosphate (cAMP), which then activates cAMP-dependent protein kinase, PKA in a mechanism classically thought to be responsible for AQP2 phosphorylation [6]. Changes in AQP2 phosphorylation status promote AQP2 trafficking to the apical plasma membrane [7–9], which results in water reabsorption from urine through AQP2 water channels to improve the dehydrated states of the body. On the other hand, renal unresponsiveness to vasopressin or defective AQP2 function in patients with congenital NDI impairs AQP2 activity and water reabsorption, resulting in polyuria.

At present, only symptomatic treatment approaches are available for congenital NDI, such as a low sodium and low protein diet, as well as the use of thiazide diuretics and nonsteroidal anti-inflammatory drugs [10]. To develop curative therapies for congenital NDI caused by V2R mutations is a challenging research proposition which is a major driving force to elucidate various regulatory mechanisms of AQP2. Well-known therapeutic strategies for congenital NDI include the rescue of V2R mutants by chemical chaperones and bypassing defective V2R signaling. In this review, we focus on activators of calcium and cAMP signaling that can increase AQP2 activity in the absence of vasopressin.

## Activators of calcium signaling

In the vasopressin signaling pathway, cAMP-induced PKA activation has been considered as a primary mechanism of AQP2 phosphorylation and trafficking [7, 11]. Recent studies have revealed that cAMP also induces intracellular calcium oscillation and both PKA and the calcium signaling pathway coordinately modulate AQP2 activity. Exchange protein directly activated by cAMP (Epac) is a key molecule that mediates cAMP and calcium signaling. Epac has two isoforms: Epac1 and Epac2. In the collecting ducts, Epac2 is mainly expressed in the apical region of all AQP2-positive cells [12]. Similar to PKA, Epac contains evolutionally conserved cAMP binding domains, which enhance calcium signaling in response to cAMP [13]. In fact, the exogenous cAMP analog 8-pCPT-2′-*O*-Me-cAMP, which selectively activates Epac, but not PKA, mimics the effects of vasopressin on calcium oscillation and promotes AQP2 trafficking toward the apical plasma membrane in isolated perfused inner medullary collecting duct (IMCD) [14, 15]. The mechanisms of Epac-mediated calcium oscillation are probably due to the release of calcium from the endoplasmic reticulum (ER) through the activation of calcium channels, such as inositol trisphosphate receptors and ryanodine receptors [16]. For this reason, calcium depletion of the ER by ryanodine or the calcium chelator BAPTA (1,2-bis(o-aminophenoxy)ethane-*N,N,N*′,*N*′-tetraacetic acid) completely inhibits vasopressin-induced calcium oscillation [17]. Although ER calcium stores are important for the initial increase in intracellular calcium concentration, extracellular calcium influx through store-operated calcium entry is also required to sustain calcium oscillation [18]. Surprisingly, ryanodine and BAPTA suppress not only calcium oscillation, but also vasopressin-induced AQP2 activation, indicating that calcium signaling exerts a critical role in AQP2 regulation in an experimental model of isolated perfused IMCD.

The precise molecular mechanisms underlying AQP2 activation by vasopressin/Epac/calcium signaling pathway remains unknown. Generally, elevation of intracellular calcium serves as a second messenger in the activation of downstream signaling molecules. Calmodulin is a calcium-binding protein that regulates various target molecules, including calmodulin-dependent protein kinases and phosphatases [19]. In addition to calcium, calmodulin plays an important role in AQP2 activation in isolated perfused IMCD. The calmodulin inhibitors W7 and trifluoperazine significantly block AQP2 trafficking and transepithelial water transport by reducing vasopressin-induced cAMP production [15]. Calmodulin may be associated with the decrease in adenylyl cyclase activity [20]. The calmodulin-dependent serine/threonine phosphatase calcineurin has also been reported to regulate AQP2. Calcineurin and AQP2 are co-localized in the intracellular vesicles of renal collecting duct cells [21, 22]. The hypertonicity-induced calcium/calmodulin/calcineurin/nuclear factor of activated T cells, cytoplasmic (NFATc) signaling pathway was reported to increase AQP2 mRNA expression in the mouse cortical collecting duct mpkCCD_cl4_ cell line [23, 24]. Calcineurin dephosphorylates NFATc in the cytosol and NFATc is subsequently translocated to the nucleus where it binds to the promoter region of the *AQP2* gene. In addition, calcineurin regulates AQP2-mediated water transport. The urine concentrating response to vasopressin is decreased in calcineurin Aα knockout mice and cyclosporine A (CyA)-treated mice [25]. CyA probably modulates AQP2 activity either directly or indirectly through impairment of the medullary osmotic gradient as a result of the inhibition of Na-K-2Cl cotransporters [26].

**Fig. 2:**
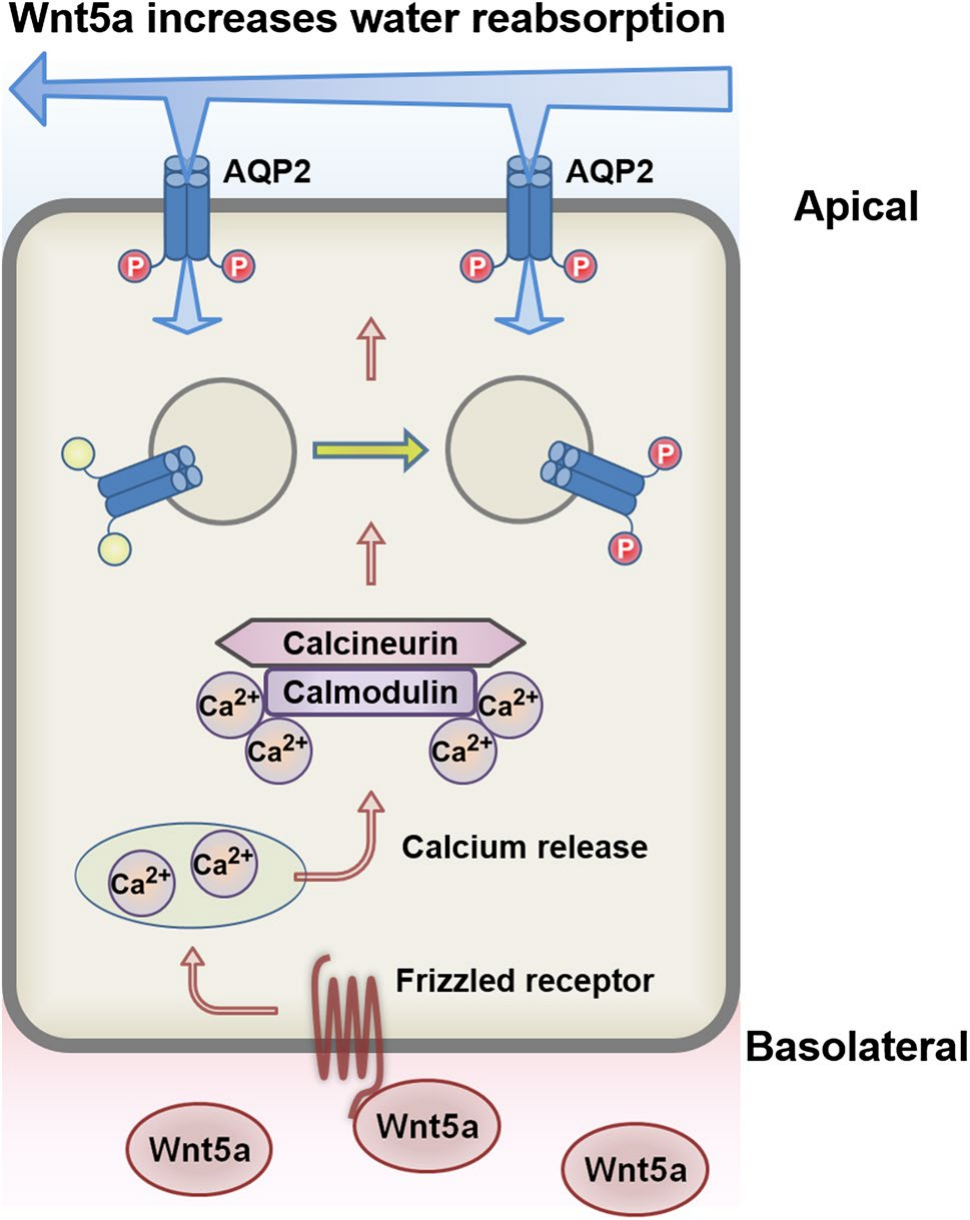
The mechanisms of urine concentration by Wnt5a. Wnt5a binds to Fzd receptors and increases intracellular calcium. The calcium-binding protein calmodulin stimulates calcineurin, which in turn, phosphorylates AQP2 and increases apical AQP2 expression. Water is then reabsorbed from urine. In addition, calcineurin increases AQP2 mRNA expression

Previous studies have suggested that the calcium signaling pathway is a major target of AQP2 activation in the treatment of congenital NDI. Therefore, we focused on the classic calcium signal transducer Wnt5a, which is a ligand of frizzled (Fzd) receptors [27–30], and found that the Wnt5a/calcium/calmodulin/calcineurin signaling pathway induced phosphorylation, trafficking, and mRNA expression of AQP2 (Fig. 2) [31]. W7 and CyA were found to totally inhibit Wnt5a-induced AQP2 activation. These effects of Wnt5a on AQP2 were examined using mpkCCD_Cl4_ cells, which are one of the most frequently used cell lines for the reliable analysis of AQP2 and exhibit endogenous expression of V2R and AQP2 [31–42]. Remarkably, contrary to the results of isolated perfused IMCD, W7 and CyA did not inhibit the effects of vasopressin on AQP2 phosphorylation in mpkCCD cells, indicating that calmodulin and calcineurin are not major regulators of vasopressin-induced AQP2 activation. The use of different experimental systems likely caused the high discrepancy in the effect of intracellular calcium on AQP2 [43]. Importantly, our results with mpkCCD cells are compatible with those obtained from clinical experience where CyA-induced NDI rarely occurred as a drug side effect. Although there are fewer effects of calcium signaling on AQP2 than those of vasopressin, Wnt5a is effective for AQP2 activation, especially in the absence of vasopressin. We demonstrated that Wnt5a increased osmotic water transport in isolated perfused cortical collecting duct (CCD) tubules of the mouse kidney and increased urine concentrating ability in a V2R-inhibited NDI mice model.

The Wnt5a signaling pathway regulates AQP2 via different mechanisms of the vasopressin/cAMP signaling pathway. Analysis of Wnt5a suggested that calcineurin is a key molecule in the activation of AQP2. The importance of calcineurin was confirmed with its direct activator arachidonic acid, which possesses vasopressin-like effects in mpkCCD cells [31]. Hence, screening for calcineurin activators is a potential therapeutic strategy to develop novel drugs for the treatment of congenital NDI.

## Elevation of cAMP concentration

cAMP is the most important key molecule in the regulation of AQP2. Significant AQP2 activation by cAMP is well established and direct cAMP activator forskolin is widely used as positive control in various assays of AQP2 activity. cAMP activation independent of defective V2R signaling is a promising therapeutic strategy for the treatment of congenital NDI and is largely divided into two methods: increased cAMP production and decreased cAMP degradation.

### G protein-coupled receptors (GPCRs) agonists

The use of GPCRs to increase cAMP production in response to their ligands has been intensively studied as a treatment option for congenital NDI. Although each GPCR has different diverse biological functions, all GPCRs share common mechanisms of signal transduction. Similar to V2R, other GPCRs also potentially increase cAMP concentrations in renal collecting ducts and activate AQP2.

The results of a TaqMan mouse GPCR array analysis revealed that IMCD cells from C57BL/6 mice express many GPCRs, including V2R [44]. The calcitonin receptor is a GPCR in renal collecting ducts. Calcitonin increases intracellular cAMP levels and the membrane accumulation of AQP2 in LLC-PK1 cells [45]. Analysis of vasopressin-deficient Brattleboro rats showed that calcitonin reduced urine flow and increased urine osmolality two-fold during the first 12 h of treatment. Angiotensin II is a ligand of the AT1 receptor, which increases AQP2 expression in the apical plasma membrane of mpkCCD cells [38]. The effects of angiotensin II on AQP2 are mediated through cAMP and calcium signaling, and are inhibited by the PKA inhibitor H89 and the calmodulin inhibitor W7. Secretin, a ligand of the secretin receptor, also has a vasopressin-like effect. Secretin increases cAMP concentrations in isolated IMCD tubule suspensions from wild-type and tamoxifen-induced V2R knockout mice [46]. In addition, secretin receptor knockout mice exhibit mild polyuria, polydipsia, and reduced renal expression of AQP2 [47]. Although GPCR agonists certainly increase intracellular cAMP levels in renal collecting ducts and activate AQP2, their effects do not persist for very long, probably due to the downregulation or desensitization of receptors [45, 46].

**Fig. 3:**
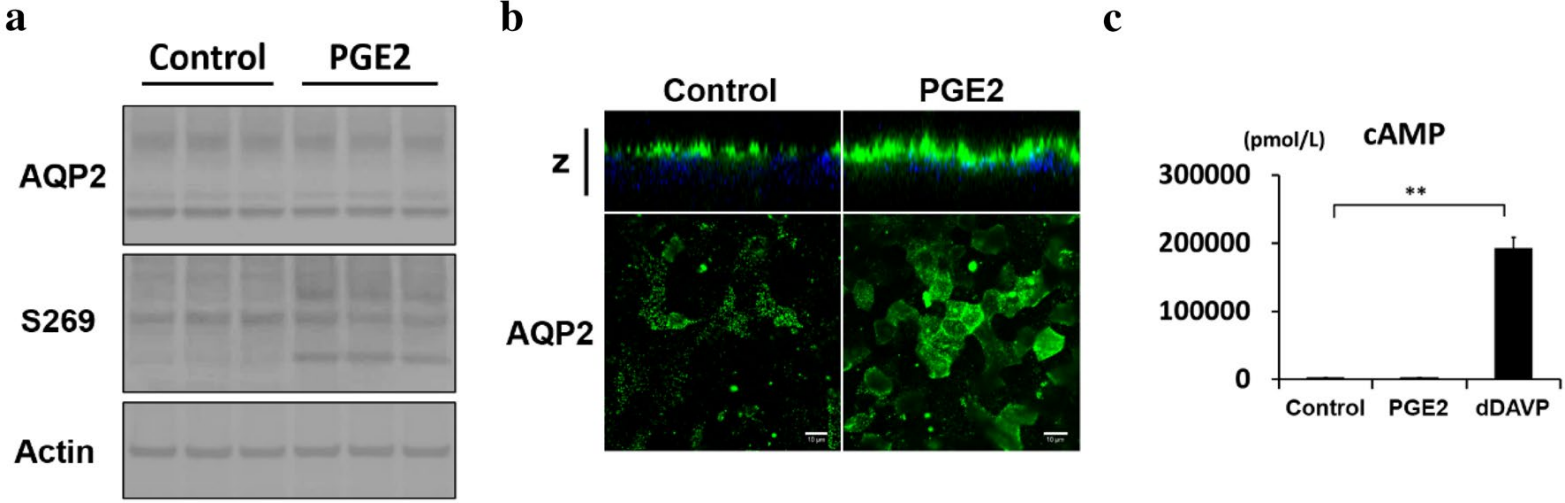
PGE2 activates AQP2 without an elevation in cAMP in mpkCCD cells. **a** PGE2-induced AQP2 phosphorylation at S269. PGE2 (10 nM) was added to the basolateral side of the mpkCCD cells for 1 h, as previously described [31]. **b** PGE2-induced AQP2 trafficking. mpkCCD cells were treated with PGE2 (10 nM) for 1 h, and the subcellular localization of AQP2 was then analyzed by immunofluorescence and confocal microscopy. The larger panels display confocal sections of the apical regions of the cells. Z-stack confocal images are shown at the top of each panel. Representative confocal images of three independent experiments are shown. Scale bars, 10 µm. **c** No significant elevation of cAMP concentration in response to PGE2. The mpkCCD cells were treated with PGE2 (10 nM) or [deamino-Cys1, *d*-Arg8]-vasopressin (dDAVP) (1 nM) for 1 h. Bars are mean values ± SD of three experiments. Asterisks indicate a significant difference as compared with the control. **p < 0.01

Prostaglandin E2 (PGE2) is a ligand of four different G protein-coupled E-prostanoid receptors: EP1–EP4. PGE2 and butaprost, selective agonists of EP2, both increased cAMP levels and AQP2 activity in MDCK cells [48]. In addition, butaprost increased urine concentrating ability in a V2R-inhibited NDI rat model [48], whereas EP4 activation by ONO-AE1-329 [44] or CAY10580 [49] increased cAMP levels and AQP2 activities in IMCD cells. The importance of EP4 in the regulation of AQP2 has also been clarified in vivo. Renal tubule-specific and collecting duct-specific EP4 knockout mice showed impaired urine concentrating abilities [49]. Moreover, subcutaneous injection of ONO-AE1-329 improved urine concentrating ability and other major manifestations, such as distension of the renal pelvis, in tamoxifen-inducible V2R knockout mice [44]. In contrast, EP4 possesses cAMP-independent effects on AQP2. In MDCK and mpkCCD cells, EP4 activation by CAY10580 increased AQP2 activity without elevating cAMP levels [50]. We also confirmed that PGE2 increased AQP2 phosphorylation and trafficking without the elevation of cAMP levels in mpkCCD cells, which endogenously express EP4, but not EP2 (Fig. 3a–c). However, the underlying mechanisms of EP4-induced AQP2 activation remains to be elucidated.

PGE2 is a lipid mediator that is not stored in cells, but rather is derived from arachidonic acid, which is released from phospholipids in the nuclear membranes of most cell types [51]. In the kidney, the conversion of arachidonic acid to PGE2 is catalyzed by the action of cyclooxygenase enzymes and two PGE synthases: cytosolic PGE synthase and microsomal PGE synthase type 1 [52]. As mentioned above, arachidonic acid directly activates calcineurin and then increases AQP2 phosphorylation, trafficking, and mRNA expression in mpkCCD cells [31]. In the arachidonic acid cascade, both arachidonic acid and PGE2 are responsible for AQP2 activity. Importantly, higher intake of omega-6 polyunsaturated fatty acids, such as arachidonic acid, appears to be safe and may reduce the risk of coronary heart disease, relative to a lower intake [53]. Hence, the arachidonic acid cascade is a potential therapeutic target for the treatment of congenital NDI.

### Phosphodiesterase (PDE) inhibitors

cAMP-PDEs degrade cAMP to AMP and decrease intracellular cAMP levels. Interestingly, PDE activity was increased in a mouse model of NDI [54, 55]. Active PDEs and defective V2R coordinate to prevent cAMP elevation in response to vasopressin. As a result of PDE activation, the PDE3 inhibitor cilostamide and the PDE4 inhibitor rolipram were found to highly restore vasopressin-induced cAMP accumulation in IMCDs of NDI mice [56]. In addition, we previously evaluated the effects of PDE inhibitors in NDI mice. First, we identified three families with frameshift mutations in *AQP2* that cause autosomal dominant NDI and then generated a disease model of knockin mice to verify the effects of PDE inhibitors [4]. Autosomal dominant NDI is characterized by a milder clinical presentation of symptoms because AQP2 tetramers composed of only wild-type AQP2 can be translocated to the cell surface, whereas those containing at least one AQP2 mutant are missorted to the basolateral membrane [5]. Rolipram increased cAMP content and promoted the translocation of wild-type AQP2 toward the apical plasma membrane. Conversely, the PDE3 inhibitor milrinone had no effect. These results suggest that rolipram is a potential therapeutic target for X-linked NDI caused by V2R mutations as well as autosomal dominant NDI. However, rolipram treatment failed to relieve the symptoms of two male patients with X-linked NDI [57]. For this reason, there are likely differences in AMP metabolism between mice and humans, thus alternative PDE4 inhibitors may be more suitable [58].

## Conclusion

Over the last two decades, the mechanisms of AQP2 regulation have been gradually clarified and many target molecules for the treatment of congenital NDI have been proposed. Although these potential therapeutic candidates, including activators of calcium and cAMP signaling, induce AQP2 activation without vasopressin *in vitro*, they failed to sufficiently increase urine concentration in vivo. Therefore, no specific pharmacological drugs have yet reached clinical application. In the development of drugs for the treatment of congenital NDI caused by V2R mutations, it may be necessary to shift focus from conventional therapeutic approaches to novel strategies that can activate AQP2 more directly and potently. At the same time, it is also important for AQP2 activation to generate a medullary osmotic gradient in the kidney because AQP2 is not a transporter, but rather a water channel. Water reabsorption occurs only after a driving force of passive water movement is created. Further studies of AQP2-activating mechanism are required to design feasible drug candidates for improvement of the excessive urine output and quality of life of NDI patients.
